# Experimental study on the ratio model of similar materials in the simulation test of coal and gas outburst

**DOI:** 10.1038/s41598-021-92880-y

**Published:** 2021-06-29

**Authors:** Peng Sun, Haitao Sun, Fujin Lin, Xuelin Yang, Wangang Jiang, Winbin Wu, Quanmin Jia

**Affiliations:** 1 State Key Laboratory of Coal Mine Disaster Dynamics and Control, College of Resources and Environmental Science, Chongqing University, Chongqing, 400044 China; 2 China Coal Technology Engineering Group Chongqing Research Institute, No. 55, Shangqiao No. 3 Village, Shapingba District, Chongqing, China

**Keywords:** Natural hazards, Engineering

## Abstract

To obtain the similar materials with specific physical and mechanical parameters and adsorption and desorption indexes used in coal and gas outburst simulation tests, pulverized coal was selected as aggregate, and sodium humate was selected as cementing agent and river sand was selected as auxiliary materials. Based on this, orthogonal tests with 6 factors and 5 levels were designed, and the tests of weighing, uniaxial compression, firmness, adsorption and desorption were carried out. The parameters such as density, uniaxial compressive strength, elastic modulus, firmness coefficient and adsorption–desorption index of similar materials with different ratios were obtained, and the sensitivity of each factor was analyzed by range analysis. The influence of various factors on the similar materials was studied, and the ratio model of similar materials was obtained. The reliability of the model was verified, and a complete method for determining the ratio model of similar materials of outburst coal was put forward. The results show that the density of the similar materials increases with the river sand content, and the uniaxial compressive strength and elastic modulus increase significantly with the pulverized coal ratio and sodium humate content, and the firmness coefficient increases linearly with the pulverized coal ratio. The adsorption constant increases linearly with the sodium humate content, while the adsorption constant b decreases linearly with the sodium humate content. The initial elution rate Δp of similar materials increases at first and then decreases with the increase of sodium humate content.

## Introduction

90% of the coal in China comes from underground mining. The average mining depth of the mines has reached 500 m, and the deepest point of some large and medium-sized coal mines has reached 1500 m, and extends downward at an average rate of 20 m every year^1,2^. With the increase of mining depth, the number of coal and gas outburst accidents increase. Many shallow coal mines are transformed into outburst mines after entering deep mining stage, and the frequency and intensity of outburst also increase significantly. This is due to that a large amount of elastic energy accumulates in coal seam under the action of high stress, and there is a large amount of gas in the coal seam. Thus, when disturbed by mining, the elastic energy and gas are released rapidly, resulting in instantaneous destruction of coal and rock structure^3–6^. Among coal mine accidents in China from 2008 to 2013, gas accidents accounted for 27% of the total deaths, ranking only second to roof accidents, and the number of deaths caused by coal and gas outburst accidents in gas accidents is nearly half^7^. Therefore, if coal and gas outburst accidents can be effectively curbed, the deaths of mine workers can be greatly reduced and the safety production of coal mine can be effectively guaranteed.

Coal and gas outburst is an extremely complex coal and gas dynamic phenomenon in coal mine production, and its prediction and prevention has always been one of the ticklish problems faced by the mining industry in the world^8,9^. In addition, most coal mines in China are characterized by low coal seam permeability, soft coal quality and complex geological conditions, which result in the frequent occurrence of coal and gas outburst^10,11^. The simulation test of coal and gas outburst is an effective means to study the mechanism of coal and gas outburst. According to the similarity principle, if the similarity model and similar materials meet the similarity criterion of the prototype, the evolution process, mechanical mechanism, dynamic effect and disaster-causing mechanism of coal and gas outburst can be simulated and reproduced^12–15^.

At present, scholars in China and abroad have obtained burgeoning volumes of research outputs through simulating experiments of coal and gas outburst by using different test materials. Some scholars have done simulation experiments on coal and gas outburst by coal briquette produced by coal briquette. Kuroiwa T carried out outburst test using cylindrical protruding device with volume cylinder. Under condition of gas pressure 0.5–0.5 °C, it was found that the larger gas pressure changed when outburst occurred, the smaller coal particle was when outburst occurred; the greater the degree of coal pulverization is, the larger gas emission quantity is^16^. Deng et al. selected coal powder with prominent coal seam to press briquette with strength IV and V without adding additives. After filling high pure gas and fully adsorbing for 36–38 h, they carried out one dimensional simulation test^17^. Tang et al. placed coal powder into a 16 cm × 16 cm × 16 cm pressure chamber of coal and gas outburst simulation instrument. After press-forming under 200 t pressure tester, pulverized coal was simulated under three-dimensional stress condition^18^. Nie et al. conducted an outburst experiment for coal seam sandwiched by roof and foor. It had a three-dimensional size of 1500 mm × 600 mm × 1000 mm with 0.5 MPa gas pressure. The outburst process was investigated by analyzing the gas pressure variation, temperature variation, outburst propagation velocity, particle size of outburst coal and energy transformation^19^.

In addition, some scholars used pulverized coal or cement to produce outstanding simulation test materials. Meng et al. produced coal samples by adding 8.1% water and coal particles with diameter of 0.1–0.2 mm. After filling gas and reaching adsorption equilibrium under two-dimensional loading, they carried out a series of stress simulation tests under different pressures. There are two typical types of failure occuring in coal sample “cracking” and “outburst”^20^. Zhang et al. conducted coal and gas outburst test in Schoczynski Mining Institute. The test simulated outburst failure process of 3 kinds of outburst coal seam, briquette and bituminous briquette under different gas pressure, established dimensionless parameter criterion for judging outburst failure of coal seam, and gave prediction formula of outburst intensity of model coal sample^21^. Ou et al. selected soft stratified coal sample below 1 mm diameter prepared by adding coal tar of different proportions under pressure 20 MPa. By using gas as adsorption gas to simulate coal and gas outburst, they obtained outburst evolution rule of different intensity coal samples^22^.

Although scholars have obtained a large number of research achievements through outburst simulation tests, it’s still not possible to physically restore the outburst phenomenon completely. The main reason is that similarity between test materials and original outburst coal is low. As an important carrier of physical simulation test of coal and gas outburst, outburst simulation similarity material directly determines physical reduction capability and representative behavior of real environment^23–25^. For example, although coal briquette directly press-formed by pulverized coal is not added with ash, its mechanical strength and firmness coefficient are generally low; although the mechanical strength and firmness coefficient of coal briquette added with cement, asphalt and other things are improved, the adding of ash content affects the adsorption and permeability of coal briquette.

Scholars in China have also conducted amount of research on the properties of coal and gas outburst simulation test materials, especially binder ratio and aggregate ratio. Kong et al. carried out experimental research on similar materials using cement and gypsum as cementing agent. Experiment results show that compressive strength and density increase with sand binder ratio and water ratio, and the cementing material ratio is a major factor to improve compressive strength of similar materials^26,27^. Liu et al. carried out an experimental study on low strength similar materials using gypsum, fly ash as cementing agent and sand as aggregate. It was found that the elastic modulus and compressive strength of similar materials have a linear relation with sand binder ratio and a power correlation with cementing agent ratio^28^. Kang et al. carried out experimental studies on similar materials of simulated raw coal using sand and pulverized coal as aggregates, and compared and analyzed the differences between the two kinds of aggregates. When pulverized coal is taken as aggregate, the material strength has a linear negative correlation with it; while sand is taken as aggregate, the material strength has a nonlinear negative correlation with it^29^. Zhang et al. carried out experiments on similar materials of outburst coal using cement as binder and pulverized coal as aggregate. It was found that there is a linear relation between the specific gravity of cement and cement sand and the uniaxial compressive strength and density of the specimens^30^.

To sum up, most research on outburst test materials and outburst simulation similar materials done by scholars in China and abroad mainly focus on the similarity of physical and mechanical properties with raw coal, and the similarity of adsorption and desorption property is rarely studied. Therefore, in order to ensure that similar materials have high similarity with the original outburst coal in terms of physical and mechanical properties and adsorption and desorption properties, a model test study on the proportion of similar materials of outburst coal was conducted in this paper. It aims at making a certain range of physical and mechanical parameters and adsorption indicators of similar materials needed for outburst simulation test, which would provide guidence for large-scale coal and gas outburst simulation test. In this paper, not only the proportioning model of similar materials for outburst coal within a certain range of physical parameters and adsorption desorption indexes is obtained, but also a complete method for determining the proportioning model of similar materials for outburst coal is proposed.

## Similarity indexes of outburst simulation test materials

Based on the analysis of the mechanical mechanism of coal and gas outburst, the similarity theory of coal and gas outburst simulation test is divided into three parts: (1) The static deformation and failure of coal in the preparation stage of outburst are similar, so it needs to meet the similarity of geometric shape, material properties, load and displacement constraints^31–33^; (2) The fragmentation of gas-bearing coal in the stage of outburst initiation and development is similar, so it needs to meet the similarity of parameters such as porosity, gas pressure and crack length^34–37^; (3) The movement of broken coal and gas flow in mining space is similar. Since the gas flow of outburst crushed coal is solid–gas two-phase flow, it needs to meet the similarity of parameters such as gas occurrence and gas emission^38–40^.

The similarity of mechanical parameters, porosity, etc. in outburst simulation test is determined by properties of similar materials. Therefore, on the premise that the outburst coal body shows the same homogeneity, using homogeneous continuous medium model theory^41–45^, and this paper derives model material similarity ratio, the model is as follows:

1 $$\begin{aligned} & \frac{\partial }{{\partial x}}\left( {K_{x} \frac{{\partial P}}{{\partial x}}} \right) + \frac{\partial }{{\partial y}}\left( {K_{y} \frac{{\partial P}}{{\partial y}}} \right) + \frac{\partial }{{\partial z}}\left( {K_{z} \frac{{\partial P}}{{\partial z}}} \right) = 2p\frac{{\partial e}}{{\partial t}} + S(p)\frac{{\partial p}}{{\partial t}} + W \\ & G\nabla ^{2} u + (\lambda + G)\frac{{\partial e}}{{\partial x}} + X - \rho \frac{{\partial ^{2} u}}{{\partial t^{2} }} = 0 \\ & G\nabla ^{2} v + (\lambda + G)\frac{{\partial e}}{{\partial y}} + Y - \rho \frac{{\partial ^{2} v}}{{\partial t^{2} }} = 0 \\ & G\nabla ^{2} w + (\lambda + G)\frac{{\partial e}}{{\partial z}} + Z - \rho \frac{{\partial ^{2} w}}{{\partial t^{2} }} = 0 \\ \end{aligned}$$

In the formula:$P = p^{2}$, $S(p) = \frac{n}{p} + \frac{{ab}}{{p(1 + bp)^{2} }}$, $K_{x}$, $K_{y}$, $K_{z}$ are permeability coefficients on the direction of three coordinate axes, $W$ is source sink term, $G = \frac{E}{{2(1 + \mu )}}$ is shear elastic modulus, $\nabla ^{2} = \frac{{\partial ^{2} }}{{\partial x^{2} }} + \frac{{\partial ^{2} }}{{\partial y^{2} }} + \frac{{\partial ^{2} }}{{\partial z^{2} }}$ is Laplace operator, $\lambda = \frac{{\mu E}}{{(1 + \mu ) + (1 - 2\mu )}}$ is Lame constant, $e = \frac{{\partial u}}{{\partial x}} + \frac{{\partial v}}{{\partial y}} + \frac{{\partial w}}{{\partial z}}$ is volume strain.

These equations are applicable for prototype (k) and model (m), given: $C_{G} = \frac{{G^{k} }}{{G^{m} }}$ is similarity ratio of shear modulus, $C_{u} = \frac{{u^{k} }}{{u^{m} }}$ is displacement similarity ratio, $C_{\lambda } = \frac{{\lambda ^{k} }}{{\lambda ^{m} }}$ is Lame similarity ratio, $C_{E} = \frac{{E^{k} }}{{E^{m} }}$ is elastic modulus similarity ratio, $C_{l} = \frac{{x^{k} }}{{x^{m} }}$ is geometric similarity ratio, $C_{e} = \frac{{e^{k} }}{{e^{m} }}$ is volume strain similarity ratio, $C_{\gamma } = \frac{{X^{k} }}{{X^{m} }}$ is bulk density similarity ratio, $C_{\rho } = \frac{{\rho ^{k} }}{{\rho ^{m} }}$ is density similarity ratio, $C_{t} = \frac{{t^{k} }}{{t^{m} }}$ is motion time similarity ratio, $C_{f} = \frac{{f^{k} }}{{f^{m} }}$ is external load similarity ratio, $C_{P} = \frac{{P^{k} }}{{P^{m} }}$ is gas pressure similarity ratio, $C_{g}$ is gravity acceleration similarity ratio and $C_{\sigma }$ is stress similarity ratio.

When the above relation is brought into the second equation of formula (1), the following formula can be obtained:

2 $$C_{G} \frac{{C_{u} }}{{C_{l} ^{2} }} = C_{G} \frac{{C_{e} }}{{C_{l} }} = C_{\lambda } \frac{{C_{e} }}{{C_{l} }} = C_{\rho } \frac{{C_{u} }}{{C_{t} ^{2} }} = C_{\gamma }$$

As it is a mathematical model of homogeneous continuous medium, $K_{x} = K_{y} = K_{z} = K$, the following functions are introduced:$C_{K} = \frac{{K^{k} }}{{K^{m} }}$ is similarity ratio of permeability coefficient, $C_{Q} = \frac{{Q^{k} }}{{Q^{m} }}$ is gas flow similarity ratio, $C_{S} = \frac{{S^{k} }}{{S^{m} }}$ is similarity ratio of gas storage coefficient and $C_{x} = \frac{{x^{k} }}{{x^{m} }} = C_{y} = \frac{{y^{k} }}{{y^{m} }} = C_{z} = \frac{{z^{k} }}{{z^{m} }} = C_{l}$ is geometric similarity ratio of 3D direction. When it is brought into the seepage Eq. (1), the following formula can be obtained:

3 $$\frac{{C_{K} C_{P} }}{{C_{x} ^{2} }} = \frac{{C_{K} C_{P} }}{{C_{y} ^{2} }} = \frac{{C_{K} C_{P} }}{{C_{z} ^{2} }} = \frac{{C_{e} }}{{C_{t} }} = C_{S} \frac{{C_{P} }}{{C_{t} }} = C_{w}$$

Through analysis of formula (2) formula (3), and in combination with geometric similarity ratio of $C_{l} = 10$ and the bulk density similarity ratio of $C_{\gamma } = 1$ between the test model and the prototype, the following relation can be derived: geometric similarity:$C_{u} = C_{l} = 10$, elastic modulus and gravity similarity:$C_{E} = C_{G} = C_{l} C_{\gamma } = 10$, stress similarity:$C_{p} = C_{\gamma } C_{l} = 10$, gas storage coefficient similarity:$C_{S} = \frac{1}{{C_{\gamma } C_{l} }} = 0.1$, seepage coefficient:$C_{K} = \frac{{\sqrt {C_{l} } }}{{C_{\gamma } }} \approx 3.2$.

Through consulting statistics of characteristic parameters of each outburst coal in Yuyang Coal Mine and combining with similar model material similarity ratio, this paper determines the characteristic parameters range of coal and outburst simulation test materials, as shown in Table 1.

**Table 1:** Characteristic parameters of outburst coal and model material.

Material	Density (g/cm^3^)	Uniaxial compressive strength (MPa)	Elastic modulus (MPa)	*f* value	Adsorption constant		Initial speed of emission (*Δp*)
					*a* (m^3^/t)	*b* (MPa^−1^)	
Outburst coal	1.21–1.72	4.3–37.8	1135–4602	0.11–0.50	15–60	0.2–2	11–39
Model material	1.21–1.72	0.43–3.78	113.5–460.2	0.11–0.50	15–60	0.2–2	11–39

## Model experiment of ratio model for outburst coal similar materials

### Experiment scheme

#### Selection of raw materials

Selection of raw materials for similar materials should conform to the following principles: (1) Easy to largely control material performance index; (2) meet prototype material characteristic requirements; (3) raw material has stable performance; (4) production process is simple; (5) Materials are safe and pollution-free^46,47^.

Raw materials of similar materials are generally composed of aggregates, binder and auxiliary materials. Combined with characteristics of model materials, M8 coal seam above 80 meshes and pulverized coal (anthracite) of 40–80 meshes from Yuyang Coal Mine are selected and used as aggregate; Sodium humate of 80–100 meshes is selected and used as cementing agent. With strong adsorption capacity, sodium humate can easily adjust adsorption and desorption index of similar materials; River sand of 0.425–0.850 mm is selected and used as auxiliary materials, which makes it easy to adjust the density of similar materials.

#### Selection of raw materials

(1) Physical and mechanical property

Orthogonal experimental method was adopted to design the experiment. Coal ratio (above 80 meshes and 40–80 meshes of pulverized coal mass ratio), sodium humate content and sand quality were selected as three factors of the orthogonal experiment. Each factor was set 5 levels respectively. Table 2 lists detail parameters.

**Table 2:** Regressional orthogonal experiment design.

Level	Pulverized coal ratio	Sodium humate content (%)	River sand content (%)
1	1:5	0.5	1
2	2:5	2.5	3
3	3:5	4.5	5
4	4:5	6.5	7
5	5:5	8.5	9

An orthogonal table of 6 factors and 5 levels was selected in the experiment. Table 3 lists the specific material ratio schemes.

**Table 3:** Ratio table of physical and mechanics parameters for similar materials of L25 (5^6^).

Experiment no	Pulverized coal ratio	Sodium humate content (%)	River sand content (%)
1	1:5	0.5	1
2	1:5	2.5	3
3	1:5	4.5	5
4	1:5	6.5	7
5	1:5	8.5	9
6	2:5	0.5	3
7	2:5	2.5	5
8	2:5	4.5	7
9	2:5	6.5	9
10	2:5	8.5	1
11	3:5	0.5	5
12	3:5	2.5	7
13	3:5	4.5	9
14	3:5	6.5	1
15	3:5	8.5	3
16	4:5	0.5	7
17	4:5	2.5	9
18	4:5	4.5	1
19	4:5	6.5	3
20	4:5	8.5	5
21	5:5	0.5	9
22	5:5	2.5	1
23	5:5	4.5	3
24	5:5	6.5	5
25	5:5	8.5	7

(2) Properties of firmness, adsorption and desorption

This paper mainly investigates the influences of the pulverized coal ratio (pulverized coal mass ratio above 80 meshes and 40–80 meshes) and the firmness coefficient of similar materials. Due to the strong adsorbability of sodium humate, under the condition of fixed pulverized coal ratio and river sand content, this paper examines the influence of different sodium humate content and adsorption and desorption indexes of similar materials. The experimental design is shown in Table 4.

**Table 4:** Ratio table of firmness coefficient, adsorption and desorption index of similar materials.

Experiment No	Firmness coefficient			Experiment No	Adsorption desorption index		
	Pulverized coal ratio	Sodium humate content (%)	River sand (%)		Pulverized coal ratio	Sodium humate content (%)	River sand (%)
1	0.2	0.5	4	6	0.4	4	0.5
2	0.4	0.5	4	7	0.4	4	2.5
3	0.6	0.5	4	8	0.4	4	4.5
4	0.8	0.5	4	9	0.4	4	6.5
5	–	–	–	10	0.4	4	8.5

### Experiment process

**Figure 1:**
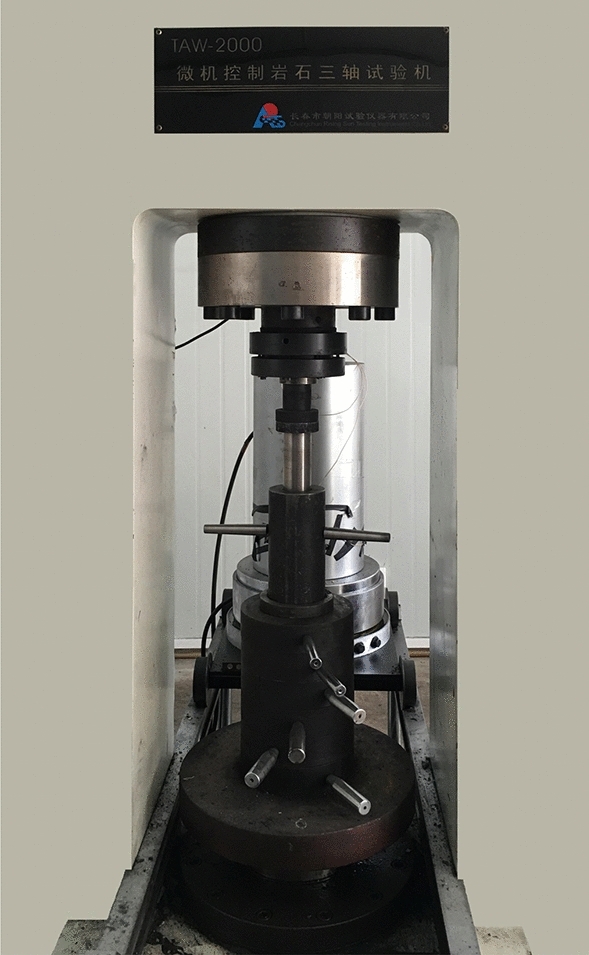
Rock triaxial testing machine controlled by TAW-2000 microcomputer.

**Figure 2:**
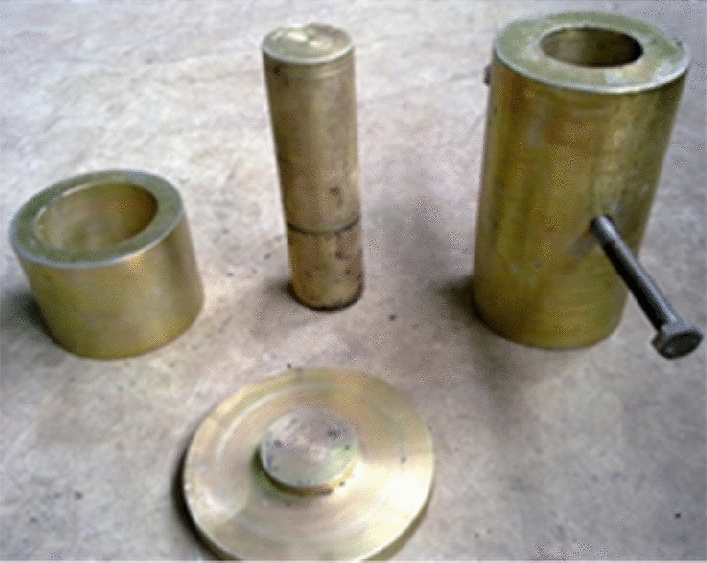
*Φ*50mm × 100 mm pressing mold.

**Figure 3:**
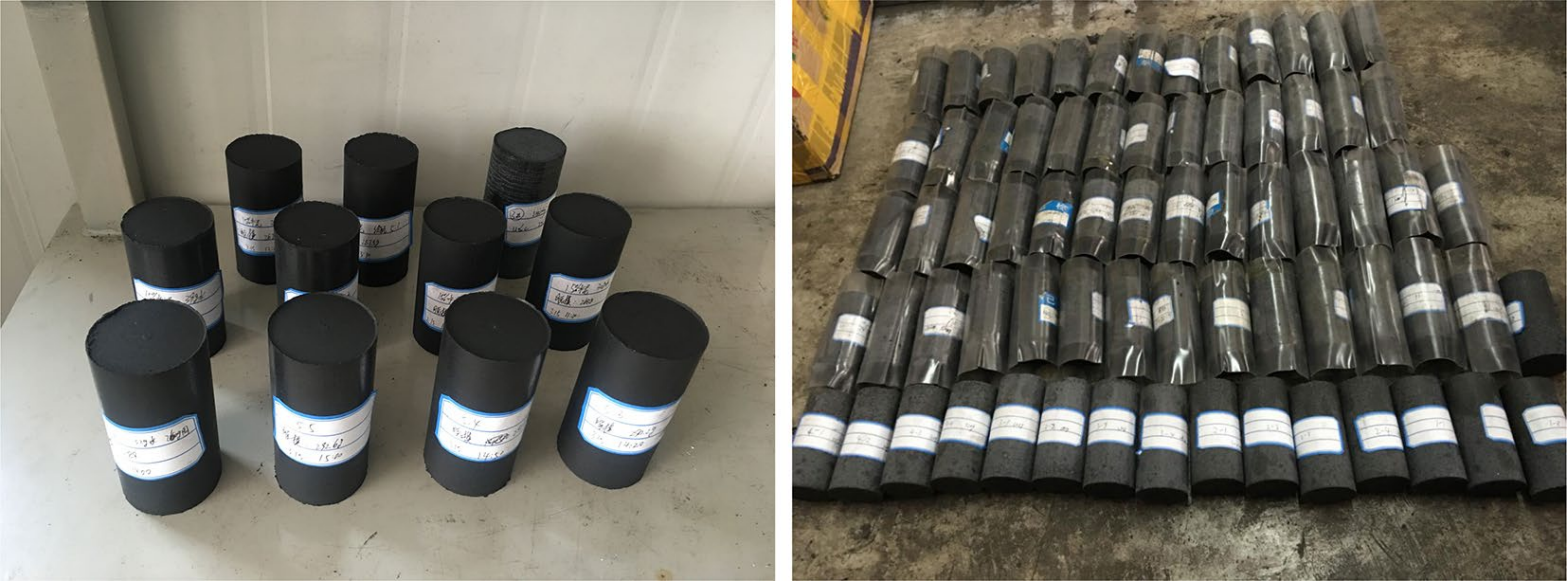
Specimens in curing.

Under the condition that the loading speed is 50 N/S, the forming stress is 20 MPa and the pressure-holding time is 15 min, the TAW-2000 microcomputer is used to control the electro-hydraulic servo rock triaxial testing machine and the mold with inner diameter of 50 mm and height of 100 mm is used to press the standard specimen according to the material ratio in Tables 3 and 4. Two new processes of stack moulding and stack retting curing are adopted in the production process. The rock triaxial testing machine is shown in Fig. 1, and the pressing mold is shown in Fig. 2, and the curing specimen is shown in Fig. 3. The production process of similar material specimens is as follows.

1. Raw material preparation: using standard sieves with different pore sizes of 0.45 mm (40 mesh) and 0.9 mm (20 mesh) to divide the crushed coal sieves into three sizes of < 40 mesh, 40–80 mesh and > 80 mesh. Then the mass of sodium humate, pulverized coal and river sand were weighed according to the experimental scheme.
2. Material mixing: mix sodium humate with 10% water and fully dissolve it, then add pulverized coal and river sand (sodium humate dry powder) into the mixer in turn, mix well, then add sodium humate aqueous solution (water), and finally stir for 2 min (if manually stirred, the mixing time should be extended for at least 5 min).
3. Heap retting and ripening: put the evenly stirred materials into plastic bags for retting. The retting time at higher room temperature generally takes 48 h, while it takes more than 72 h at lower room temperature.
4. Prepare the test mold: in order to reduce the friction between the test piece and the inner wall of the cylinder and facilitate the demoulding, apply silicone oil evenly on the inner wall of the pressing mold cylinder.
5. Charging and tamping: take the smaller inner diameter of the cylinder as the lower end and combine it with the tray, then use the funnel to load the mixed raw materials into the mold, and use the rubber hammer to gently tap the pressure bar to tamp the raw materials. The tamping degree can make the pressure bar stable on the raw materials and not easy to slide.
6. Press molding: put the mold with tamping materials in the center of the test bench, and set the operating parameters and start pressing, and then keep it for a period of time after reaching the predetermined molding pressure.
7. Demoulding and marking: place the demoulding sleeve in the center of the discus of the testing machine, and lay a certain thickness of soft cloth on the bottom of the demoulding sleeve, then put the pressing die upside down on the demoulding sleeve for pressure demoulding, and finally label and number the demoulded specimen.
8. Curing: the pressed specimen is exposed to the air and cured indoors for a certain period of time.

### Experiment results

Size measurement, weighing, uniaxial compression test, firmness, adsorption and desorption tests were carried out on 25 groups of standard specimens in Table 5 and 9 groups of standard specimens in Table 6 (two standard specimens were pressed in each group). The uniaxial compressive strength, elastic modulus, density, firmness coefficient, adsorption constant and initial velocity of diffusion were measured, and the average values of the measured data are shown in Tables 5 and 6.

**Table 5:** Orthogonal test results of similar materials.

Experiment No	Uniaxial compressive strength (MPa)	Elastic modulus (MPa)	Density (g/cm^3^)
1	1.335	117.45	1.308
2	1.485	133.65	1.342
3	1.598	144.05	1.364
4	1.633	162.20	1.378
5	1.599	152.55	1.392
6	1.518	146.15	1.344
7	1.587	148.50	1.350
8	1.655	153.60	1.356
9	1.753	185.05	1.386
10	1.910	192.65	1.328
11	1.698	158.00	1.382
12	1.746	180.10	1.39
13	1.733	173.35	1.407
14	2.085	206.55	1.342
15	1.867	184.60	1.365
16	1.838	174.45	1.412
17	1.789	172.00	1.425
18	2.201	218.85	1.349
19	1.925	194.15	1.366
20	2.383	272.65	1.384
21	1.867	188.90	1.438
22	1.963	204.35	1.372
23	2.197	233.10	1.396
24	2.527	302.00	1.404
25	2.459	279.90	1.413

**Table 6:** Firmness coefficient and adsorption–desorption index of similar materials.

Experiment No	Material ratio	Parameter	Material ratio	Parameter		
	Pulverized coal ratio	Firmness coefficient (*f*)	Pulverized coal ratio	Adsorption constant		Initial velocity of diffusion (*ΔP*)
				*a*	*b*	
1	0.2	0.13	0.4	25.6248		1.7734
2	0.4	0.17	0.4	27.4399		1.7205
3	0.6	0.19	0.4	28.6718		1.6884
4	0.8	0.23	0.4	30.5426		1.3084
5	–	–	0.4	31.5511		1.3356

Comparing the test results of Table 5, Table 6 and Table 1, it is found that the configured density range of similar materials is [1.308, 1.438] ⊆ [1.21, 1.72] (model material density); the range of uniaxial compressive strength is [1.335, 2.527] ⊆ [0.43, 3.78] (model material uniaxial compressive strength); the range of elastic modulus is [117.45, 302.00] ⊆ [113.5, 460.2] (model material elastic modulus); the range of firmness coefficient is [0.13, 0.23] ⊆ [0.11, 0.50] (model material firmness coefficient); the range of adsorption constant *a* is [26, 32] ⊆ [15, 60] (model material adsorption constant a); the range of adsorption constant range *b* is [1.3, 1.8] ⊆ [0.2, 2] (model material adsorption constant b); the range of diffusion initial velocity is [16, 22] ⊆ [11, 39] (model material diffusion initial velocity). Therefore, it can ensure that the prepared similar materials have good similarity with outburst coal in physical and mechanical properties, firmness, adsorption and desorption properties.

### Discussion

#### Analysis of the influence of physical and mechanical properties

(1) Sensitivity analysis of various factors

The factors that affect the density, uniaxial compressive strength and elastic modulus of the specimen in the orthogonal test results are calculated at each level, as shown in Table 7. For the density of similar materials, the range of river sand content is the largest, which shows that river sand content has the strongest controlling effect on the density of similar materials, followed by the ratio of pulverized coal to coal, and finally the content of sodium humate. For the uniaxial compressive strength and elastic modulus of similar materials, the sensitivities of various factors are highly consistent, and the range of sodium humate content and pulverized coal ratio is much larger than that of river sand content, but the strongest controlling effect is the pulverized coal ratio, followed by sodium humate content, and finally the river sand content.

**Table 7:** The range of each level of each factor.

Factor	Density (g/m^3^)	Uniaxial compressive strength (MPa)	Elastic modulus (MPa)
Pulverized coal ratio	0.048	0.489	66.59
Sodium humate content	0.009	0.422	52.244
River sand content	0.073	0.107	18.264

**Figure 4:**
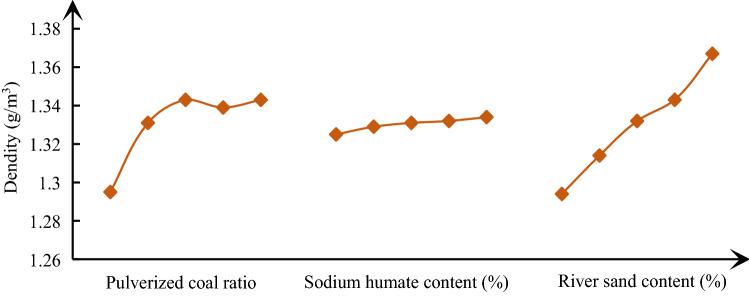
Effect curve of similar material density.

**Figure 5:**
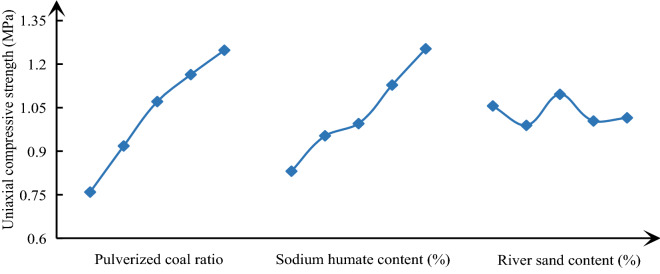
Effect curve of uniaxial compressive strength of similar materials.

**Figure 6:**
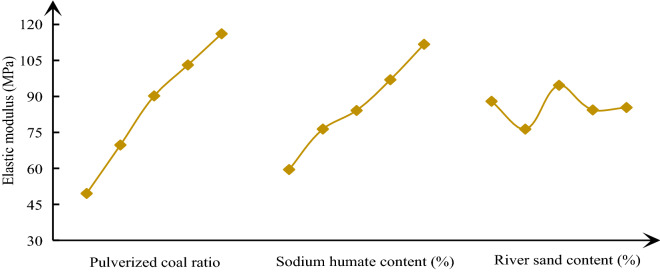
Effect curve of elastic modulus of similar materials.

In order to analyze the influence law of various factors on similar material parameters, it is necessary to calculate the mean value of each factor at each level, and then use Origin software to make a visual analysis diagram of the influence of various parameters on similar material parameters, the results are shown in Figs. 4, 5, 6. With the increase of pulverized coal ratio, the density begins to show an approximately linear increasing trend, and then gradually tends to be flat. The change of cement content has little effect on density; The change of river sediment proportion has the greatest impact on the density, showing a linear increasing relationship. The sensitivity of various factors of density from large to small is river sand content > pulverized coal ratio > sodium humate content. From Fig. 5, with the increase of the proportion of pulverized coal and the specific gravity of cementing agent, the uniaxial compressive strength presents an approximate linear relationship. However, the change of specific gravity of river sediment has no effect on the uniaxial compressive strength, and the curve has no obvious change rule. The sensitivity of each factor of uniaxial compressive strength from large to small is powder coal ratio > sodium humate content > river sand content. From Fig. 6, with the increase of the proportion of pulverized coal and the specific gravity of cementing agent, the elastic modulus also presents an approximate linear relationship. However, the effect of the specific gravity of river sediment on the elastic modulus is not obvious. The order of sensitivity of each factor of elastic modulus from large to small is powder coal ratio > sodium humate content > river sand content.

(2) Multiple linear regression analysis

Through the above sensitivity analysis of various factors, there is an obvious linear relation between each factor and some parameters of similar materials. Multiple linear regression analysis was carried out by using SPSS software. Let pulverized coal ratio be the equal of $K_{1}$, sodium humate content $K_{2}$, river sand content $K_{3}$, density $M_{1}$, uniaxial compressive strength $M_{2}$, elastic modulus $M_{3}$. The regression equations were obtained as follows:

4 $$\begin{aligned} & M_{1} = 0.065K_{1} + 0.008K_{3} + 1.295 \\ & M_{2} = 0.844K_{1} + 0.053K_{2} + 1.11 \\ & M_{3} = 120.285K_{1} + 8.062K_{2} + 78.704 \\ \end{aligned}$$

Under the condition that the pulverized coal ratio, the sodium humate content and the river sand content are known, the density, uniaxial compressive strength and elastic modulus of similar materials can be calculated through formula (4). However, in order to obtain the raw material ratio of similar materials with a specific parameter, the formula (4) is solved and the following empirical formula is obtained:

5 $$\begin{aligned} & K_{1} = 16.41M_{2} - 0.12M_{3} - 11.13 \\ & K_{2} = - 280.24M_{2} + 1.97M_{3} + 156.3 \\ & K_{3} = 125M_{1} - 133.33M_{2} + 0.98M_{3} - 71.44 \\ \end{aligned}$$

Under the condition that the density, uniaxial compressive strength and elastic modulus of similar materials are known, the pulverized coal ratio, sodium humate content and river sand content of similar materials can be calculated through formula (5). The ratio of pulverized coal $K_{1} \in \left[ {0,\infty } \right]$, the sodium humate content $K_{2} \in \left[ {0,1} \right]$ and the river sand content $K_{3} \in \left[ {0,1} \right]$ in formula (5). When calculating the material ratio by using the above equation, if the calculation result exceeds the range of appeal value, it shows that the selection of similar materials for this kind of raw material configuration under this process condition does not meet the experiment requirements, and it is necessary to select other raw materials or change the process conditions.

#### Analysis of the influence of firmness and adsorption and desorption performance

(1) The firmness of similar materials

The firmness of coal differs from the strength of coal. As a comprehensive index of the ability to resist external damage determined by various properties of coal, it is also one of the main identification indexes of outburst coal seam in the detailed rules for Prevention and Control of Coal and Gas Outburst. Therefore, it is listed as one of the important indexes in the test of similar materials of outburst coal^48,49^.

6 $$f = 0.16K_{1} + 0.1$$

**Figure 7:**
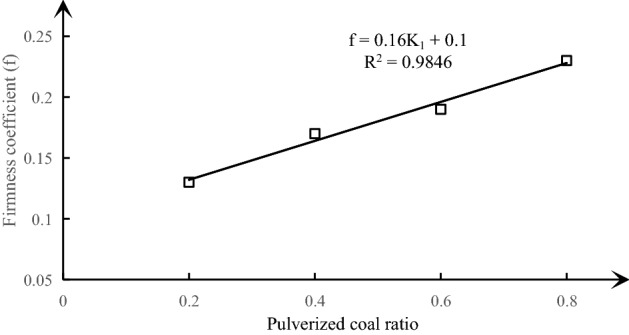
Curve of pulverized coal ratio and firmness coefficient under the condition of fixed sodium humate and river sand content.

A visual analysis diagram of the effect of different pulverized coal ratio on the firmness coefficient of similar materials under the condition of fixed sodium humate and river sand content is made according to Table 6, as shown in Fig. 7. Figure 7 shows that the firmness coefficient of similar materials increases linearly with the increase of pulverized coal ratio, and the fitting degree R^2^ of the relational formula is as high as 0.9846, showing good fitting effect. The relational formula (6) is obtained.

(2) Adsorption and desorption properties of similar materials

**Figure 8:**
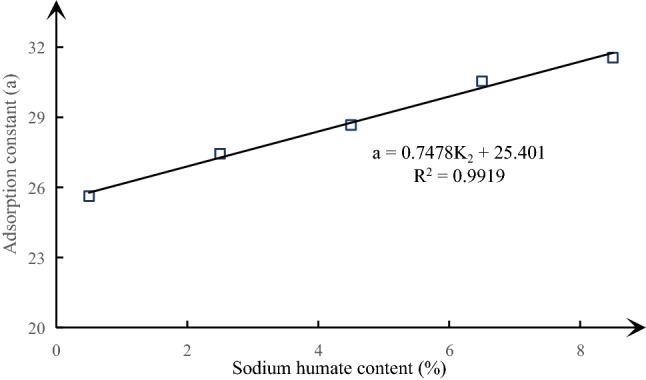
The curve of sodium humate and adsorption constant a under the condition of fixed pulverized coal ratio and river sand content.

**Figure 9:**
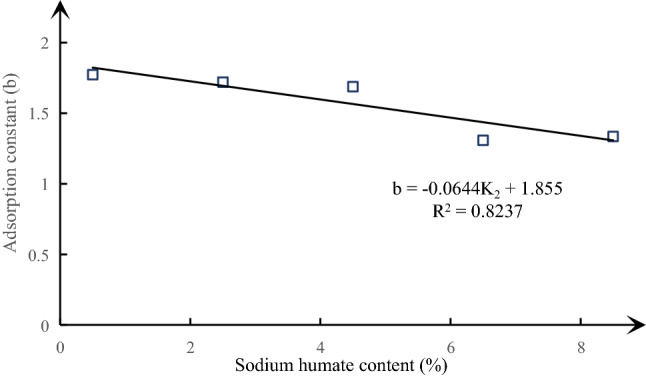
The curve of sodium humate content and adsorption constant b under the condition of fixed pulverized coal ratio and river sand content.

**Figure 10:**
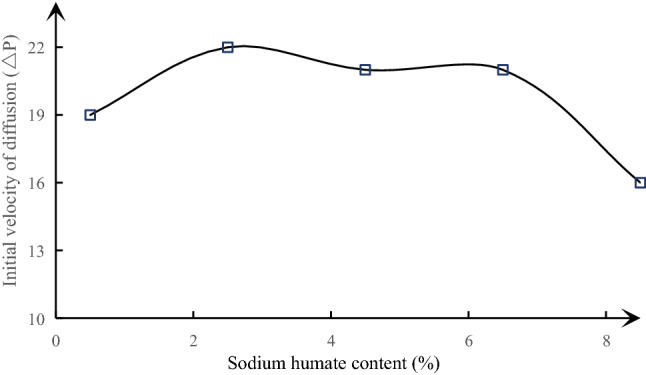
The curve of sodium humate and initial diffusion velocity (ΔP) under the condition of fixed pulverized coal ratio and river sand content.

A visual analysis diagram of the effect of different sodium humate content on the adsorption constant and initial diffusion velocity of similar materials is made according to Table 7, as shown in Figs. 8, 9 and 10. It can be seen from the above figure that the adsorption constant *a* of similar materials increases gradually with the increase of sodium humate content, but the adsorption constant *b* decreases gradually, showing a good linear relationship. The linear fitting degree R^2^ between adsorption constant *a* and sodium humate content is as high as 0.9919, the fitting degree R^2^ of adsorption constant *b* is 0.8237. The relationship obtained by fitting is shown in formula (7). The initial diffusion velocity of similar materials increases at first and then decreases with the increase of sodium humate content.

7 $$\begin{aligned} & a = 0.7478K_{2} + 25.401 \\ & b = - 0.0644K_{2} + 1.855 \\ \end{aligned}$$

## Verification analysis of the matched model of similar materials

The specific method of verification analysis: select three groups of similar material parameters from Table 4 and calculate the material ratio of each group of parameters through formula (5), and make the standard specimen with these three groups of raw materials under the same standard pressing process, then determine the parameters of the specimen, and then compare and analyze the parameters with the three groups of similar material parameters selected, and verify the ratio model through the difference of the two parameters. It can be seen from Figs. 5 and 6 that the effects of pulverized coal ratio, sodium humate content and river sand content on uniaxial compressive strength and elastic modulus are highly consistent, so only density and uniaxial compressive strength are considered in this verification test. The selected three groups of parameters and the calculated material ratio are shown in Table 8.

**Table 8:** Raw material ratio of similar materials.

Experiment no	Parameter		Material ratio		
	Density (g/m^3^)	Uniaxial compressive strength (MPa)	Pulverized coal ratio	Sodium humate (%)	River sand (%)
1	1.308	0.753	0.2	1.3	0.1
2	1.390	1.055	0.5	3.8	7.7
3	1.396	1.454	0.8	7.2	5.8

The same raw materials and abrasive tools were selected to press the standard specimens according to the three groups of materials in Table 8 in the same environment and the same standard production process, and the density and uniaxial compressive strength of the specimens were measured in the same curing time. The measured and analyzed data are shown in Table 9.

**Table 9:** Comparison of experiment values and original values of density and uniaxial compressive strength of similar materials.

Experiment no	Density (g/m^3^)			Uniaxial compressive strength (MPa)		
	Original values	Experiment values	Relative deviation (%)	Original values	Experiment values	Relative deviation (%)
1	1.308	1.352	3.36	1.335	1.104	17.30
2	1.390	1.386	0.29	1.746	1.437	17.70
3	1.396	1.377	1.36	2.197	1.908	13.15

In addition, under the matching conditions of pulverized coal ratio 0.4, sodium humate content 0.5% and river sand content 4%, the set of parameters of similar materials with firmness coefficient 0.17, adsorption constant a 25.6248, adsorption constant b 1.7734 and initial diffusion velocity 19 were also selected from Tables 6 and 7. The ratio of materials back calculated by formula (6) and formula (7) is 0.4 and the content of sodium humate is 0.3%. In the case of fixed river sand content of 4%, the specimen was pressed with the same standard production process, and the firmness coefficient and adsorption and desorption index were determined after curing for 15 days (see Table 10), and then compared with the original index to verify the formula.

**Table 10:** Comparison of adsorption constant, firmness coefficient and initial diffusion velocity of similar materials between experiment values and original values.

Experimental data	Raw material ratio			Parameter			
	Pulverized coal ratio	Sodium humate content (%)	River sand content (%)	Adsorption constant		Initial velocity of diffusion (*ΔP*)	Firmness coefficient (*f*)
				*a*	*b*		
Original values	0.4	0.5	4	25.6248	1.7734	19	0.17
Experiment values	0.4	0.3	4	24.5461	1.8325	16	0.15
Relative deviation	–	–	–	4.21%	3.33%	15.8%	11.8%

In the model experiment of similar materials, there is no clear specification for the allowable error of the characteristic parameters of similar materials. According to the Basic Performance Test Method of Building Mortar^50^, the difference between the maximum or minimum value and the average value of the material performance index shall not exceed 20%, so the maximum difference between the experiment value and the original value of the similar material shall not exceed 20%, which is also used as a standard to measure the regression equation and the experiment value. As can be seen from Tables 9 and 10, the relative deviations of similar material density, uniaxial compressive strength, firmness coefficient, adsorption constant, initial velocity of diffusion and original parameters calculated by regression Eq. (5), formula (6) and formula (7) are all less than 20%. Therefore, the empirical formula obtained from this experimental study can be effectively used to configure the model materials in the simulation test of coal and gas outburst.

## Conclusions

1. By using the method of orthogonal design, 25 groups of material ratio schemes are designed based on three factors: sodium humate content, pulverized coal ratio and river sand content. Each factor is set at 5 levels. Similar materials with density range of 1.308–1.438 g/cm^3^, uniaxial compressive strength range of 1.335–2.527 MPa and elastic modulus range of 117.45–302.00 MPa were obtained under different material ratio conditions.
2. The sensitivity of various factors to the physical and mechanical properties of similar materials is analyzed by range analysis. The effect on the density of similar materials is in the following order: river sand content > pulverized coal ratio > sodium humate content, and the effects on uniaxial compressive strength and elastic modulus of similar materials are as follows: pulverized coal ratio > sodium humate content > river sand content.
3. The influence of various factors on the parameters of similar materials is studied. The density of similar materials increases with the increase of river sand content, increases first and then remains at a certain level with the increase of pulverized coal ratio, and the uniaxial compressive strength and elastic modulus of similar materials increase significantly with the increase of pulverized coal ratio and sodium humate content. However, there is no obvious change with the increase of river sand content, and the firmness coefficient of similar materials increases linearly with the increase of pulverized coal ratio (linear equation: $f = 0.16K_{1} + 0.1$); the adsorption constant a of similar materials increases linearly with the increase of sodium humate content (the linear equation: $a = 0.7478K_{2} + 25.401$), adsorption constant b decreases linearly with the increase of sodium humate content (the linear equation: $b = - 0.0644K_{2} + 1.855$); the initial diffusion velocity of similar materials increases at first and then decreases with the increase of sodium humate content.
4. The multiple linear regression analysis of the experimental data is carried out by using SPSS software, and the empirical formula of the ratio of similar materials is obtained. The verification test shows that the similar material ratio calculated by the empirical formula and the similar material parameters prepared under the same standard production process meet the test requirements.
5. By standardizing the similarity index of coal and gas outburst test materials, the raw materials of similar materials, the mold of similar materials and the pressing process of similar material specimens, this paper puts forward a complete method for determining the ratio model of similar materials of outburst coal based on the empirical formula of the ratio of similar materials of outburst coal.

## References

[1] Xie HP, Zhou HW, Xue DJ, Wang HW, Zhang R, Gao F (2012). Research and thinking on deep mining and limit mining depth of coal. J. China Coal Soc..

[2] Cheng YP, Wang L, Zhang XL (2011). Environmental impact of coal mine methane emissions and responding strategies in China. Int. J. Greenhouse Gas Control.

[3] Hu WY (2008). Research Status and Development Trend of Deep Coal Resources and Their Development Technical Conditions.

[4] Zhao H, Xiong ZQ, Wang W (2010). Main problems and countermeasures of deep mining in mine. Coal Eng..

[5] Li HM, Fu K (2006). Main technical problems and countermeasures in deep mining of coal mine. J. Min. Saf. Eng..

[6] Lu J, Zhang DM, Huang G, Li X, Gao H, Yin GZ (2020). Effects of loading rate on the compound dynamic disaster in deep underground coal mine. Int. J. Rock Mech. Min. Sci..

[7] Jing GX (2014). Analysis on the law of coal mine gas accidents in China from 2008 to 2013. J. Saf. Environ..

[8] Fu JH, Cheng YP (2007). Present situation and prevention countermeasures of coal and gas outburst in coal mines in China. J. Min. Saf. Eng..

[9] Yang X, Wen G, Lu T (2020). Optimization and field application of CO2 gas fracturing technique for enhancing CBM extraction. Nat. Resour. Res..

[10] Liu C, Yin GZ, Li MH (2019). Deformation and permeability evolution of coals considering the effect of beddings. Int. J. Rock Mech. Min. Sci..

[11] Hu QT, Wen GC (2013). Mechanical Mechanism of Coal and Gas Outburst.

[12] Xu T (1982). Similarity Theory and Model Test.

[13] Li SC, Li QC, Wang HP, Yuan L, Zhang YQ, Xue JH, Zhang B, Wang J (2018). Development of large-scale real three-dimensional coal and gas outburst quantitative physical simulation test system. J. China Coal Soc..

[14] Duan M, Jiang C, Xing H (2020). Study on damage of coal based on permeability and load-unload response ratio under tiered cyclic loading. Arab. J. Geosci..

[15] Yuan L, Wang W, Wang HP, Zhang B, Liu ZZ, Yu GF, Zuo YJ (2020). Simulation test system of coal and gas outburst induced by roadway driving. J. China Univ. Min. Technol..

[16] Kuroiwa T, Tashiro T (1960). Experimental study on coal pulverization and gas emission in a moment of out-bursts of gas and coal. J. Jpn. Min..

[17] Deng QF, Luan YX, Wang YA (1989). Simulation test of coal and gas outburst. Coal Mine Saf..

[18] Tang JP, Pan YS, Yang L (2013). Simulation experimental study on coal and gas outburst under three-dimensional stress. Chin. J. Rock Mechan. Eng..

[19] Nie BS, Ma Yk HuST, Meng JQ (2019). Laboratory study phenomenon of coal and gas outburst based on a mid-scale simulation system. Sci. Rep..

[20] Meng XY, Ding YS, Chen L, Bai RS, Tan QM (1996). Two-dimensional simulation experimental study on coal and gas outburst. J. China Coal Soc..

[21] Zhang JG, Wei FQ (2002). Outburst simulation test of gas-bearing coal. Min. Saf. Environ. Prot..

[22] Ou JC (2012). Simulation Experimental Study on the Evolution Process of Coal and Gas Outburst.

[23] Li SG, Zhao B, Zhao PX, Yang EH, Xu PY (2019). Study on gas adsorption characteristics of coal-rock gas solid-gas coupling similar materials. J. Min. Saf. Eng..

[24] Wang HP, Li QC, Yuan L, Li SC, Xue JH, Zhu HY, Duan CR, Wang SG (2018). Coal and gas outburst simulation test research and development of similar materials and characteristic analysis of briquette. J. Min. Saf. Eng..

[25] Dai LC (2020). Experimental research on the proportion and mechanical properties of outburst briquette. Min. Saf. Environ. Protect..

[26] Kong LQ, Sun JM (2007). Experimental study on similar material ratio of simulated coal. Opencast Min. Technol..

[27] Li BF, Ren YK, Qi LW, Chang L (2011). Experimental study on orthogonal proportion of low strength similar materials for coal and rock mass. Coal Eng..

[28] Liu LL, Wang HL, Liu JB, Chen SJ (2014). Orthogonal proportion test of low strength similar materials. J. Liaoning Univ. Eng. Technol..

[29] Kang XT, Huang Y, Deng BZ, Han PB (2015). Experimental study on similar materials simulating raw coal. J. Northeastern Univ..

[30] Zhang ST, Dai LC, Wang B, Cao J (2015). Experimental study on similar material ratio of simulated coal and gas outburst. Coal Sci. Technol..

[31] Yin GZ, Jiang CB, Wang JG, Xu J, Zhang DM, Huang G (2016). A new experimental apparatus for coal and gas outburst simulation. Rock Mech Rock Eng..

[32] Zhang CL, Xu J, Yin GZ, Peng S, Li Q, Chen YX (2019). A novel large-scale multifunctional apparatus to study the disaster dynamics and gas flow mechanism in coal mines. Rock Mech. Rock Eng..

[33] Fan CJ, Li S, Luo MK, Du WZ, Yang ZH (2017). Coal and gas outburst dynamic system. Int. J. Min. Sci. Technol..

[34] Lu YY, Wang HY, Xia BW, Li XH, Ge ZL, Tang JR (2017). Development of a multi-functional physical model testing system for deep coal petrography engineering. Rock Mech Rock Eng..

[35] Tu QY, Cheng YP, Guo PK, Jiang JY, Wang L, Zhang R (2016). Experimental study of coal and gas outbursts related to gas-enriched areas. Rock Mech Rock Eng..

[36] Guo BH, Li YZ, Jiao F, Luo T, Ma Q (2018). Experimental study on coal and gas outburst and the variation characteristics of gas pressure. Geomech. Geophys. Geo-energ. Geo-resour..

[37] Bin ZA, Jiang X, Peng SJ, Geng B, Yan FZ (2019). Test system for the visualization of dynamic disasters and its application to coal and gas outburst. Int. J. Rock Mech. Min. Sci..

[38] Zhang QH, Yuan L, Wang HP, Kang JH, Li SC, Xue JH, Zhou W, Zhang DM (2016). Establishment and analysis of similarity criterion for physical simulation of coal and gas outburst. J. China Coal Soc..

[39] Dai LC, Zhang ST, Cao J (2018). Discussion on geometric parameters of similar model of coal and gas outburst. Coal Mine Safety..

[40] Cao J, Sun HT, Dai LC, Sun DL, Wang B, Miao FT (2018). Simulation study on dynamic effect of coal and gas outburst. J. China Univ. Min. Technol..

[41] Zhao YS (2010). Multi-field Coupling in Porous Media and Its Engineering Response.

[42] Li SG, Zhao PX, Lin HF, Xiao P, Wei ZY (2015). Experimental study on similar material characteristics of “solid-gas” coupling physical simulation of coal and gas. J. China Coal Soc..

[43] Li SC, Feng XD, Li SC, Li LP, Li GY (2010). Development and application of new solid-fluid coupling similar materials. J. Rock Mech. Eng..

[44] Ping G, Shugang C, Zunguo Z, Yi Li, Yanbao L, Yong Li (2012). Solid-gas coupling mathematical model and numerical simulation of gas-bearing coal. J. China Coal Soc..

[45] Yin GZ, Wang DK, Zhang DM, Huang G (2008). Study on solid-Gas Coupling dynamic Model and numerical Simulation of Gas-bearing Coal. J. Geotech. Eng..

[46] Gu DZ (1995). Similar Materials and Similar Models.

[47] Wu J (1987). Study on microstructure and surface characteristics of outburst coal. J. China Coal Soc..

[48] Zhao XS, Hu QT, Zou YH, Kang JN (2007). Principle and application of rapid determination of firmness coefficient of deep coal. J. China Coal Soc..

[49] State Administration of Coal Mine Safety Supervision (2009). Prescribed Reading for the Prevention and Control of Coal and Gas Outburst.

[50] Ministry of Construction of the People’s Republic of China (1990). Test Method for Basic Properties of JGJ70-90 Building Mortar.

